# Mapping gene by early life stress interactions on child subcortical brain structures: A genome-wide prospective study

**DOI:** 10.1002/jcv2.12113

**Published:** 2022-11-16

**Authors:** Koen Bolhuis, Rosa H. Mulder, Casper Louk de Mol, Serena Defina, Varun Warrier, Tonya White, Henning Tiemeier, Ryan L. Muetzel, Charlotte A. M. Cecil

**Affiliations:** 1 Department of Child and Adolescent Psychiatry/Psychology, Erasmus MC-Sophia, Rotterdam, The Netherlands; 2 Department of Pediatrics, Erasmus MC-Sophia, Rotterdam, The Netherlands; 3 Department of Neurology, MS Center ErasMS, Erasmus MC, Rotterdam, The Netherlands; 4 Department of Psychiatry, University of Cambridge, Cambridge, UK; 5 Department of Radiology and Nuclear Medicine, Erasmus MC, Rotterdam, The Netherlands; 6 Department of Social and Behavioral Sciences, Harvard TH Chan School of Public Health, Boston, Massachusetts, USA; 7 Department of Epidemiology, Erasmus MC, Rotterdam, The Netherlands; 8 Molecular Epidemiology, Department of Biomedical Data Sciences, Leiden University Medical Center, Leiden, The Netherlands

**Keywords:** early life stress, gene-environment interaction, genome-wide association study, MRI, psychopathology

## Abstract

**Background:**

Although it is well-established that both genetics and the environment influence brain development, they are typically examined separately. Here, we aimed to prospectively investigate the interactive effects of genetic variants–from a genome-wide approach–and early life stress (ELS) on child subcortical brain structures, and their association with subsequent mental health problems.

**Method:**

Primary analyses were conducted using data from the Generation R Study (*N* = 2257), including genotype and cumulative prenatal and postnatal ELS scores (encompassing life events, contextual risk, parental risk, interpersonal risk, direct victimisation). Neuroimaging data were collected at age 10 years, including intracranial and subcortical brain volumes (accumbens, amygdala, caudate, hippocampus, pallidum, putamen, thalamus). Genome-wide association and genome-wide-by-environment interaction analyses (GWEIS, run separately for prenatal/postnatal ELS) were conducted for eight brain outcomes (i.e., 24 genome-wide analyses) in the Generation R Study (discovery). Polygenic scores (PGS) using the resulting weights were calculated in an independent (target) cohort (adolescent brain cognitive development Study; *N* = 10,751), to validate associations with corresponding subcortical volumes and examine links to later mother-reported internalising and externalising problems.

**Results:**

One GWEIS-prenatal stress locus was associated with caudate volume (rs139505895, mapping onto *PRSS12* and *NDST3*) and two GWEIS-postnatal stress loci with the accumbens (rs2397823 and rs3130008, mapping onto *CUTA, SYNGAP1,* and *TABP*). Functional annotation revealed that these genes play a role in neuronal plasticity and synaptic function, and have been implicated in neuro-developmental phenotypes, for example, intellectual disability, autism, and schizophrenia. None of these associations survived a more stringent correction for multiple testing across all analysis sets. In the validation sample, all PGS_genotype_ were associated with their respective brain volumes, but no PGS_GxE_ associated with any subcortical volume. None of the PGS associated with internalising or externalising problems.

**Conclusions:**

This study lends novel suggestive insights into gene-environment interplay on the developing brain as well as pointing to promising candidate loci for future replication and mechanistic studies.

## Introduction

Subcortical brain structures show differential developmental patterns over time (Raznahan et al., 2014) and variations in their volumes are associated with a wide range of child and adult psychiatric disorders. Subcortical brain structures are under considerable genetic influence as demonstrated by recent twin heritability studies (Strike et al., 2019) and a genome-wide association study (GWAS) (Satizabal et al., 2019), which has so far identified 48 significant loci and 199 associated genes. These genes are implicated in a wide range of neurodevelopmental regulatory processes, including synaptic signalling, axonal transport and neural apoptosis. Furthermore, genes associated with subcortical brain structures are also modestly associated with psychiatric phenotypes such as schizophrenia, attention-deficit/hyperactivity disorder (ADHD), autism spectrum disorder (ASD), bipolar disorder, as well as with cognition (Ohi et al., 2020; Satizabal et al., 2019; Smith et al., 2021).

Besides genetic influences, many previous studies have found that subcortical brain structures are also influenced by environmental risk factors from as early as gestation, which have been shown to affect their growth and development (Lupien et al., 2009; Modabbernia et al., 2021; Tost et al., 2015). Indeed, stressors both in utero (e.g., prenatal maternal depression) and postnatally (e.g., poor family functioning, childhood adversities) have been associated with variation in subcortical brain volumes (McLaughlin et al., 2019; Quinlan et al., 2020; Xerxa et al., 2021; Zou et al., 2019).

Taken together, evidence to date points to a key role of both genetic and environmental factors in subcortical brain development. However, few studies have examined gene-environment interactions on the brain (Gao et al., 2019) and, of these, most have looked at candidate genes, primarily focussing on monoamine neurotransmitter (Meer et al., 2015; Reed et al., 2018) and neuroendocrine-related (Malhi et al., 2020; Wesarg et al., 2021) pathways. Other studies have employed polygenic scores (PGS) based on GWAS of psychiatric disorders and subsequently examined interactive effects with specific environmental stressors on brain structure (Acosta et al., 2020; Bolhuis et al., 2019; French et al., 2015). Although PGSs capture broader genetic influences than candidate gene approaches, it remains unclear to what extent individual genetic factors across the genome interact with environmental factors. One notable exception is a recent study on a sample of 184 neonates, reporting that genome-wide-by-environment interaction (GWEIS) models outperformed GWAS-only and environment-only models to best explain variation in amygdala and hippocampus volume and microstructure (Ong et al., 2019), although which specific single nucleotide polymorphism (SNPs) or environments were implicated in these interactions was not reported. In short, as most studies used a cross-sectional design, focused on candidate genes or PGS, and lacked prospective assessments of ELS, it remains unclear to what extent genes and environment interact to shape variation in subcortical brain volumes in childhood and downstream risk for psychopathology. This is important considering that the aetiology of childhood-onset mental disorders remains largely unknown and integrative studies are considered a crucial next step to map gene-environment interplay on brain phenotypes (Arango et al., 2021; Solmi et al., 2021).

The primary aim of this study was to perform a gene-environment interaction analysis, in which we investigated the interactive effects of common genetic variants–from a genome-wide approach–and early life stress (ELS) on child subcortical brain volumes, as well as their associations with subsequent internalising and externalising problems in emerging adolescence. Discovery analyses were embedded in the Generation R Study, to our knowledge the largest, single site, paediatric population-based study including comprehensive prospective measures of prenatal and postnatal ELS spanning pregnancy to childhood, genotyping and neuroimaging. Validation was performed in the adolescent brain cognitive development (ABCD) Study, an independent sample with a larger sample size, but fewer (and retrospectively assessed) ELS measures. Individual hits from GWAS and GWEIS in the Generation R study were carried forward in the ABCD Study for validation. In addition, PGS were calculated to test associations with subcortical brain volumes and behavioural outcomes in the independent ABCD Study.

## Methods

### Study populations

Primary analyses were conducted using data from the Generation R Study, a prospective cohort study from the general population of Rotterdam, the Netherlands (Kooijman et al., 2016). Participants were included when genotype, cumulative scores of prenatal and postnatal ELS (birth to age 10 years) and neuroimaging data at age

10 years were available. Genotyping was done separately for two independently genotyped subsamples, that is, GENR3 (*n* = 1722) and GENR4 (*n* = 535), which were meta-analysed (combined *n* = 2257). Validation analyses were performed in the US-based ABCD Study (Casey et al., 2018), an independent study including over 10,000 youth aged 9–10 years. First, discovery and validation analyses were done in all participants, which included mixed ethnic-genetic ancestry. Sensitivity analyses were performed in participants of European ancestry to account for genetic stratification effects.

Full details on genotyping, neuroimaging assessment, and ELS measurements of the Generation R and ABCD samples are provided in the Supporting Information 1. In short, genotyping was performed with Illumina HumanHap 610 or 660 quad chips for the Generation R Study, and with the Smokescreen™ Genoyping array for the ABCD Study. Quality control and genotype imputation were harmonised across the two cohorts. Neuroimaging was performed using 3-Tesla scanners in both cohorts. Segmentation of T_1_-weighted images was performed using FreeSurfer (v6.0) with the extraction of seven subcortical volumes per hemisphere. In the Generation R Study, prospective assessments on a wide range of environmental and psychosocial risks were used to calculate cumulative scores of ELS, spanning two developmental periods, that is, during pregnancy (prenatal ELS score) and from birth to age 10 years (postnatal ELS score, up to the age of the MRI assessment). Briefly, ~100 stress items were selected, dichotomised into absent (=0) or present risk (=1), and assigned to one of the following domains: life events, contextual risk, parental risk, interpersonal risk, and direct victimisation (only available postnatally). Dichotomised risks were summed to create domain scores, and domain scores were subsequently summed and standardised within periods to obtain prenatal and postnatal ELS scores. These ELS scores are based on earlier studies, have been used in multiple cohorts, and have been found to prospectively associate with mental health and cognitive outcomes (Cecil et al., 2014; Clayborne et al., 2021; Rijlaarsdam et al., 2016; Schuurmans et al., 2022). In ABCD, ELS measures were retrospectively assessed when the child was on overage 9 years, including both prenatal and postnatal exposures.

### Statistical analyses

Our primary linear regression analyses were performed with an adapted version of the *GEM* software package (Pan et al., 2016) in R version 4.0.5 (R Core Team, 2015). Three analysis sets were conducted to test for (1) G, (2) E and (3) GxE effects (see Figure S1 for analysis overview). For each of these sets, 8 brain outcomes were tested and an analysis set-wide Bonferroni correction was applied to correct for multiple comparisons. This resulted in (1) G set: eight GWAS analyses (genome-wide significance: *p* < 5e^−8^; set-wide Bonferroni-correction: *p* < 5e^−8^/8 = 6.25e^−9^); (2) E set: 16 associations (eight for prenatal ELS and eight for postnatal ELS; set-wide Bonferroni-correction: *p* < 0.05/16 = 3.12e^−3^); and (3) GxE set: 16 GWEIS analyses (eight for GxPrenatal ELS, and eight for GxPostnatal ELS; genome-wide significance: *p* < 5e^−8^; set-wide Bonferroni-correction *p* < 5e^−8^/16 = 3.12e^−9^). The results from GENR3 and GENR4 were meta-analysed using inverse variance-weighted fixed effects models with the METAL software (Willer et al., 2010). Significant results from the *Gmodel* and *GxEmodel* were followed forward for characterisation using functional mapping and annotation of genome-wide association studies (Watanabe et al., 2017) to identify lead/independent SNPs, annotated genes and enriched biological functions as well as previously identified phenotypic associations based on the GWAS Catalogue (MacArthur et al., 2017).

Subsequently, SNPs identified in GWAS/GWEIS at a genome-wide threshold of significance were assessed for validation in the ABCD Study. Further, the GWAS summary statistics of the G and GxE analysis sets in the Generation R cohort were carried forward to calculate PGS for external validation in the ABCD Study. The polygenic risk score under continuous shrinkage (PRS-CS) method (Ge et al., 2019) was used to infer posterior effect sizes of SNPs using a highdimensional Bayesian regression framework to capture polygenic variation. These were calculated separately for each subcortical volume outcome as well as separately for GWAS, GWEIS-prenatal stress, and GWEIS-postnatal stress results, resulting in three sets of PGS for eight brain outcomes. Pearson correlations between all PGS_genotype_, PGS_GxE-prenatal_, PGS_GxE-postnatal_ were calculated in the ABCD Study, based on weights from the Generation R study GWAS (i.e., eight PGSgenotype) and GWEIS (i.e., eight PGScxE-prenatal, eight PGS_GxE-postnatal_). Validation analyses proceeded in two steps. First, PGSs were examined in association with their respective brain outcome in the ABCD Study, for example, the association of PGS_genotype-accumbens_ with accumbens volume. These analyses were adjusted for sex, age, scanner type, site, intracranial volume, and four principal components of genetic ancestry. Analyses using the PGS_GxE_ were additionally adjusted for PGS_genotype_ and cumulative pre- or postnatal ELS scores to parse out the *GxEmodel* effects over and above the effects of *Gmodel* and *Emodel.* Second, if a significant association was found in the previous analysis step, PGS were next examined to test for associations with later internalising or externalising symptoms, for example, the association of PGS_GxE-prenatal-accumbens_ with internalising problems. Third, if an association emerged between (a) PGS and brain outcome as well as (b) PGS and behavioural outcome, statistical mediation was performed to assess whether brain volume mediated the association between the PGS and behaviour outcome. Again, multiple-testing correction was applied using the Bonferroni method (*p* < 0.05/24 = 2.08e^−3^).

Previously it was shown that population stratification in genetic analyses due to multi-ethnicity in the Generation R Study can be adequately controlled for by the use of genetic principal components (Medina-Gomez et al., 2015). To confirm this in the current study, we performed sensitivity analyses by rerunning analyses in samples selected for European genetic ancestry (both Generation R and ABCD cohorts).

## Results

### Sample characteristics

In this sample, 2257 participants were included for the meta-analysis (Table 1), comprising *n* = 1722 and *n* = 535 participants from GENR3 and GENR4, respectively. In total, 1049 (60.9%) and 341 (63.7%) participants were of European ancestry in GENR3 and GENR4, respectively, resulting in an *N*_combined_ = 1390 (61.6%) individuals. In the ABCD Study, *N* = 10,749 individuals were included for validation analyses, of whom 55.1% were of European genetic ancestry. Correlations between study variables are shown in Tables S1 and S2.

### *Gmodel*: Individual effects of genotype on subcortical structures

**Figure 1:**
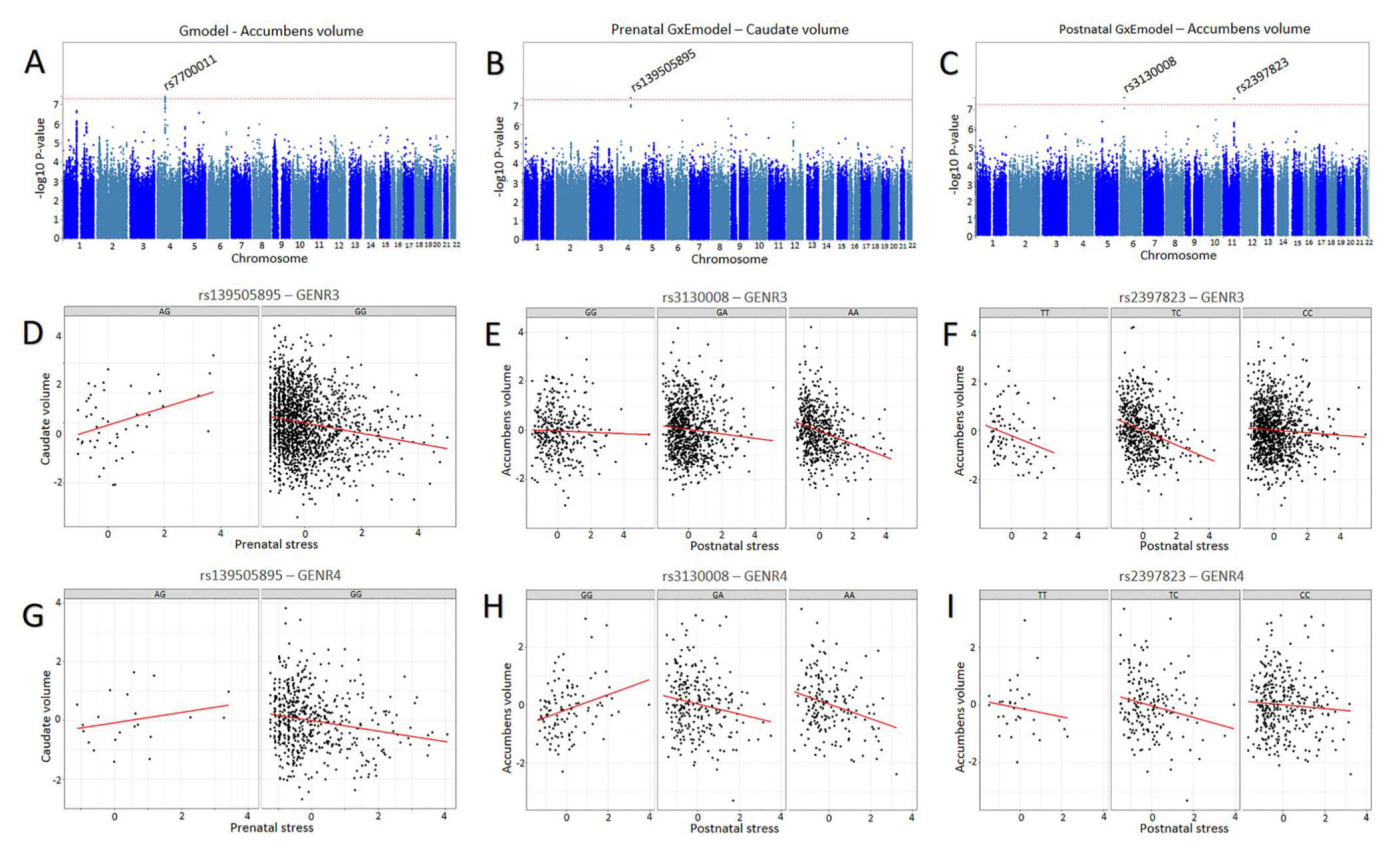
Results from the genome-wide (GWAS) and genome-wide by environment interaction analyses (GWEIS). Upper panel: (A) Manhattan plots for the GWAS for the accumbens, (B) GWEIS-prenatal stress for the caudate and (C) GWEIS-postnatal stress for the accumbens. Lower panels: interaction graphs for the GWEIS-significant findings in GENR3 (middle panel) and GENR4 (lower panel). (D/G) Across both GENR3 and GENR4 for rs13905895, prenatal early life stress (ELS) associated with lower caudate volume in carriers of the GG allele, but with higher volume in AG carriers. (E/H) For rs3130008, negative associations between postnatal ELS and accumbens volume were steepest for AA carriers compared to GA and GG carriers. (F/I) For rs2397823, negative associations between postnatal ELS and accumbens volume were steeper in the less common TT carriers compared to TC and CC carriers.

After genome-wide correction, one locus–rs7700011 on chromosome 4–was associated with bilateral accumbens volume (Table 2, Figure 1A and Table S3; *p* = 3.83e^−8^).This was no longer significant at the stricter analysis set-wide threshold. Rs7700011 is located in an intergenic region and did not map onto any genes. No other loci showed GWAS-significant associations.

### *Emodel*: Individual effects of prenatal and postnatal stress on subcortical structures

Prenatal ELS was associated with lower intracranial (Tables 1 and S4; *β* = –0.08, 95% CI –0.12; –0.04, *p* = 1.12e^−4^) and caudate volumes (*β* = –0.06, 95% CI –0.10; –0.02, *p* = 0.004). Postnatal ELS was associated with lower intracranial (*β* = –0.10, 95% CI –0.13; –0.06, *p* = 1.19e^−7^) and accumbens volumes (*β* = –0.05, 95% CI –0.09; –0.02, *p* = 0.006). Prenatal or postnatal ELS were not related with any other subcortical volumes after multiple-testing correction, although associations were generally negative.

### *GxEmodel*: Interactive effects on subcortical brain structures

#### Prenatal stress × genotype

The Manhattan plot for the GWEIS of prenatal stress is displayed in Figure 1B. One locus–rs139505895 (chromosome 4)–interacted with prenatal stress to predict bilateral caudate volume (*p* = 4.02e^−8^) as shown in Table 2 (also see as well as Figure 1D,G for scatter plot of interaction effect). This locus was no longer significant at the more conservative threshold. The SNP maps onto *PRSS12* and *NDST3* genes. *PRSS12* encodes for the neurotrypsin protein secreted from neuronal cells and has been implicated in synaptic plasticity, learning, memory and cognitive impairment. *NDST3* shows enhanced expression in the brain and variants mapped to this gene have been identified as GWAS-significant hits for multiple psychiatric traits (e.g., schizophrenia, neuroticism, worry), educational attainment and exposure to childhood maltreatment based on GWAS Catalogue results. Top ranked SNPs from the GxE-prenatal-model across brain regions are displayed in Table S5. No other loci showed genome-wide significant GWEIS effects.

#### Postnatal stress × genotype

The Manhattan plot for the GWEIS of postnatal stress is displayed in Figure 1C. After genome-wide correction, rs3130008 (chromosome 6) and rs2397823 (chromosome 11) interacted with postnatal stress to predict bilateral accumbens volume (*P* = 2.06e^−8^ and *P* = 2.28e^−8^) as shown in Table 2 (see Figure 1E,F,H,I for scatter plots of interaction effect). These loci were no longer significant at the more conservative threshold. The annotated genes for rs3130008 include *CUTA, SYNGAP1,* and *TABP* genes. *SYNGAP1* shows enhanced expression in the brain, specifically in horizontal and bipolar neuronal cells, where it has been implicated in synaptic plasticity, learning and memory. Results from the GWAS Catalogue indicate that the rs3130008 locus mapped to *SYNGAP1* has been identified as a GWAS hit for episodic memory as well as multiple psychiatric disorders, including schizophrenia and ASD. rs2397823 corresponds to an intergenic locus. Top ranked SNPs from the GxE-prenatal-model across brain regions are displayed in Table S6. No other loci showed GWEIS-significant interaction effects.

### Validation analyses in ABCD

**Figure 2:**
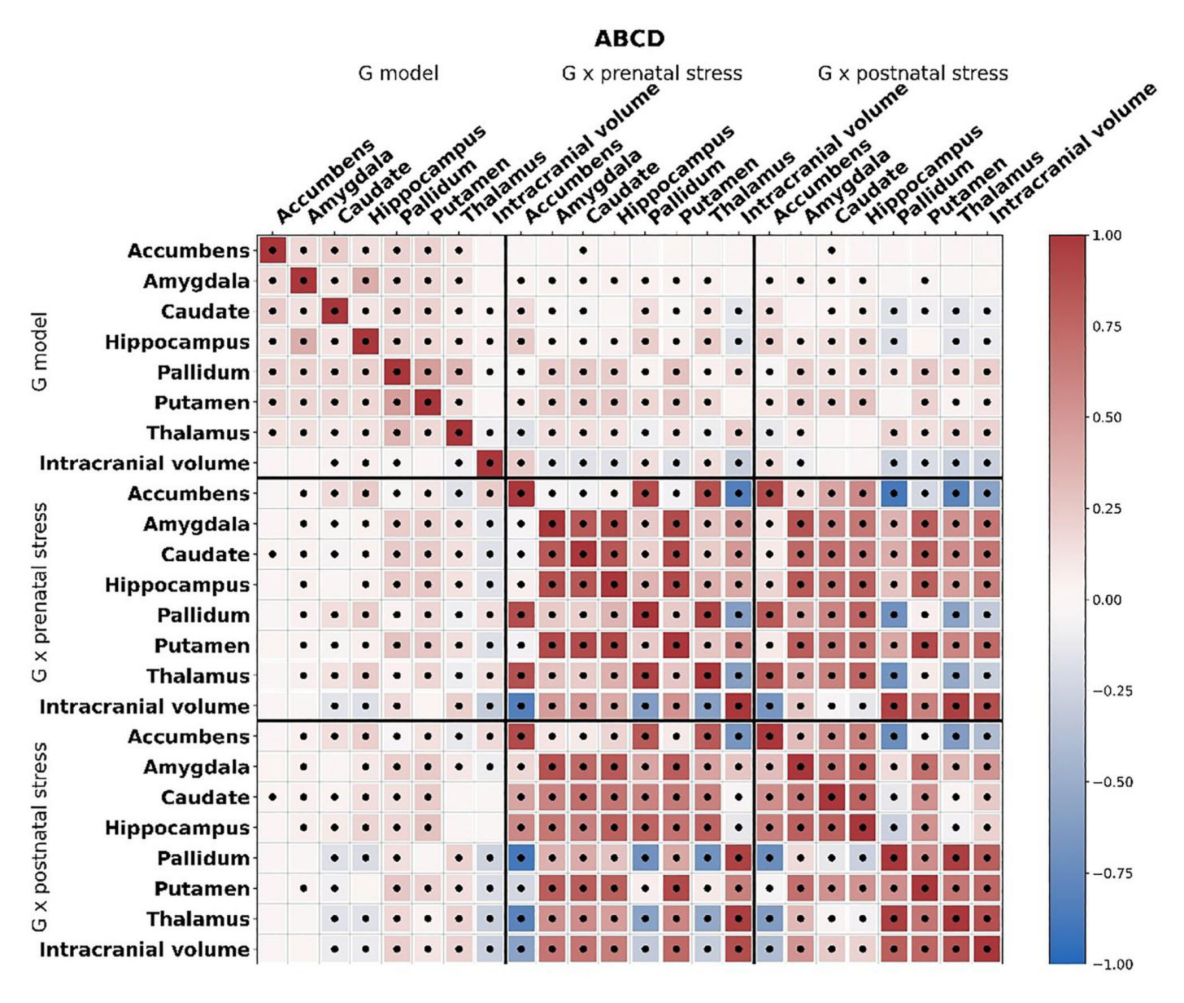
Heatmap of correlations between the genome-wide association (PGSgenotype) and genome-wide by environment interaction analyses (PGSGxE-prenatal, PGSGxE-postnatal) derived polygenic scores for each brain outcome in the ABCD Study. Red indicates a positive correlation and blue indicates a negative correlation, dots indicate *p*-values < 0.05. ABCD, adolescent brain cognitive development

Of the four genome-wide significant SNPs identified in Generation R, three were not present in the ABCD sample due to poor imputation quality. The remaining SNP, that is, rs3130008 (chromosome 6) was tested for validation in ABCD. This SNP showed a consistent pattern of associations with accumbens volume in interaction with postnatal ELS as in Generation R, although effects were of weaker magnitude (Figure S2; *P* = 0.072). PGS_genotype_ correlated modestly with both PGS_GxE-prenatal_ and PGS_GxE-postnatal_, indicating that GWAS and GWEIS-results may capture partially different signals (Figure 2). In ABCD, all PGS_genotype_ were associated with larger volumes of their respective brain structure in ABCD after multiple testing correction (Table 3). No PGS_GxE_ derived from the GWEIS model associated with brain outcomes after Bonferroni-correction for multiple testing. When testing associations of PGS_genotype_ with psychopathology, no association survived multiple-testing correction (Table S7).

### Sensitivity analyses

GWEIS and GWAS results were re-run in participants of European genetic ancestry in the Generation R discovery sample, yielding comparable results (Table S8). Similarly, PGS analyses re-run in participants of European genetic ancestry of the ABCD Study yielded comparable results with betas in the same direction (Table S9).

## Discussion

To the best of our knowledge, this is the first study to explore gene-environment interactions on child subcortical brain volumes using a genome-wide approach. We studied over 2000 children from a population-based birth cohort to characterise interactions between common genetic polymorphisms and ELS on subcortical brain volumes at age 10 years. We highlight three key findings.

First, we found suggestive evidence that variation at three genetic loci interacted with ELS to predict subcortical brain volume. Specifically, rs139505895 (chromosome 4) interacted with prenatal stress to predict caudate volume; rs3130008 (chromosome 6) and rs2397823 (chromosome 11) interacted with postnatal stress to predict accumbens volume, although these did not survive more stringent multiple-testing correction for the total number of genome-wide analyses performed. These loci map onto genes (i.e., *PRSS12, NDST3, CUTA, SYNGAP1,* and *TABP*) that play a role in synaptic plasticity, learning and memory, and have been implicated in neuro-developmental disorders such as schizophrenia and autism. Second, our analyses of main effects showed that both prenatal and postnatal ELS associate with lower intracranial volume and several subcortical volumes, whereas genome-wide significant genetic main effects were detected in the accumbens. Third, PGS_genotype_ derived from the discovery sample were all found to associate with their respective brain volume in an independent sample, providing validity for this genetic approach in a population-based sample of children. None of the PGS associated with internalising or externalising problems.

In spite of the importance of both genetic and environmental factors on brain development, few studies have examined how these may interact to shape brain structure in childhood (Gao et al., 2019; Hyde, 2015; Hyde et al., 2011). Efforts to map gene-environment interactions on the brain have so far been complicated by the need for large samples with well-characterised and ideally prospectively measured environmental exposures, genome-wide genetic data, and neuroimaging.

In this study, we identified three suggestive GWEIS hits: rs139505895 interacted with prenatal stress to predict caudate volume and rs3130008 and rs2397823 interacted with postnatal stress to predict accumbens volume. It should be noted that these associations did not meet a more stringent threshold of significance, although this threshold assumes independence between the analyses performed and may thus be considered overly conservative given the interrelatedness among the brain outcomes as well as between prenatal and postnatal ELS scores. Nevertheless, findings should be interpreted with caution, especially as these suggestive hits have not been reported in previous (candidate-gene) GxE-studies of the brain. Positional mapping of rs139505895 implicate the *PRSS12* and *NDST3* genes, which have recently been identified in a GWAS of maltreatment (Warrier et al., 2021). Interestingly, we found that ELS was negatively associated caudate volume, but only for individuals with the GG genotype of the SNP, suggesting differential biological mechanisms of risk. Another top hit, rs3130008, for which the negative association between postnatal stress and accumbens volume was most pronounced for A-allele carriers, mapped onto the *SYNGAP1* gene, which plays a role in neuronal plasticity and has been implicated by previous GWAS studies in schizophrenia (Goes et al., 2015; Ikeda et al., 2019) and ASD (Autism Spectrum Disorders Working Group of The Psychiatric Genomics Consortium, 2017). Future mechanistic studies will be needed to clarify the biological mechanisms underlying these GxE associations, including incorporating additional layers of molecular data (e.g., epigenetic and gene expression data). Of interest, none of the loci that we identify in the current study overlap with those from the most recent GWAS of subcortical structures (Satizabal et al., 2019). A reason for this could be that our study focused on children as opposed to adults. Furthermore, GWEIS-analyses may capture a (partially) distinct signal compared to GWASs. Indeed, our results indicate modest correlations between PRS derived from the GWEIS and GWAS models, suggesting that genetic variants that may show the strongest main effects on brain structure may not be those that are most sensitive to environmental exposure. Future studies will be needed to explore the molecular pathways by which gene-environment interaction in these loci occur and how they may modulate downstream neurodevelopment, behaviour, and psychiatric risk.

Based on the GWAS-analyses and GWEIS-analyses results from the Generation R samples, PGS were calculated for independent validation in the ABCD cohort. Importantly, PGS_genotype_ were consistently positively associated with their respective brain structure: for example, higher scores of the PGS_genotype_ for accumbens was associated with higher accumbens volume in the independent sample. Notwithstanding the relatively low power to detect genome-wide significant hits in such a modest discovery sample, this finding lends a degree of validity and generalisability of these genetic findings for subcortical structures in paediatric samples. Unlike the PGS_genotype_ findings, the PGS_GxE_ were less clearly associated with outcomes, and none survived multiple testing correction. These associations were generally in the negative direction, which we infer to reflect genetic influences on smaller subcortical brain volumes in the presence of ELS (Figure S3). However, the direction of associations is particularly challenging to disentangle in PGS_GxE_-analyses. Furthermore, the power to detect interactions is generally lower than the power to detect genetic main effects (Werme et al., 2021), requiring even larger sample sizes for GWEIS. Interestingly, PGS_genotype_ only correlated moderately with PGS_GxE-prenatal_ and PGS_GxE-postnatal_, suggesting that the PGSGxE capture partly distinct signals than genotype alone.

Overall, our findings lend novel insights into gene-environment interactions on child subcortical brain structure, which may help to generate hypotheses for future research investigating potentially sensitive periods of neurodevelopmental risk. Evidence of gene-environment interaction on neurodevelopment may also aid the interpretation of the clinically heterogeneous and developmentally-sensitive presentations of psychopathology, which continues to receive little attention in child and adolescent mental health practice and research (Thapar & Riglin, 2020). At the same time, the PGS (PGS_GxE_ or PGS_genotype_) were not consistently associated with measures of psychopathology in our validation sample. While this may reflect power issues, it is also possible that polygenic signal for brain volume is not predictive of mental health outcomes, which has been observed earlier in adults (Ohi et al., 2020). It should also be noted that here we focussed exclusively on common child internalising and externalising symptoms. It would be interesting to extend analyses to the outcomes implicated by our top GWEIS loci (i.e., schizophrenia, cognition and autism).

This study presents a number of strengths, including its prospective design, comprehensive measures of pre- and postnatal ELS, and validation in an independent sample. Furthermore, our findings were consistent across participants of European genetic ancestry and those of mixed genetic ancestry, highlighting the importance of conducting genetic association studies in samples that are not only mainly of European ancestry. However, several limitations should be noted. First, the discovery analyses were done in a relatively small sample for GWAS and GWEIS. An alternative strategy would be to use the ABCD Study for discovery analyses to prioritise data quantity, but we opted for the use of prospective comprehensive assessments of prenatal and postnatal ELS in our GWEIS-analyses. Increased sample sizes in conjunction with detailed phenotyping of ELS will result in greater power to detect interaction effects at the genome-wide significant level (Cai et al., 2020; Kolossa & Kopp, 2018; Werme et al., 2021). Second, we used a cumulative score of ELS, which does not capture the severity of individual stressors as well as potential interactions between risk factors (Evans et al., 2013). Third, only retrospective assessments of ELS were available in the ABCD study, which did not fully correspond with the ELS-measures in the Generation R sample, precluding a more thorough replication.

In conclusion, this study is the first to employ a genome-wide approach to investigate gene-environment interactions on subcortical brain structures in children. Three suggestive loci were identified that in interaction with ELS were associated with subcortical brain structural volume, and mapped to genes that have previously been implicated in neuroplasticity and psychiatric conditions such as schizophrenia and ASD. Genetic main effects were validated in an independent sample but much larger studies are needed to increase power to detect more gene-environment interaction effects and potential associations with neurodevelopmental outcomes. While our findings provide suggestive support for gene-environment interaction effects on the developing brain beginning in utero, these should be interpreted with caution as they did not survive more stringent correction for multiple testing. As such, findings will need to be replicated in larger (multi-cohort) studies. We hope that our findings will generate novel hypotheses for future mechanistic research to elucidate biological pathways underlying neurodevelopment and possible developmental windows of risk for psychopathology.

## Figures and Tables

**Table 1:** Descriptive characteristics

	GENR3 (*N* = 1722)	GENR4 (*N* = 535)	ABCD (*N* = 10,749)
Age at MRI assessment in years	10.16 (0.60)	10.04 (0.56)	9.91 (0.63)
Sex, proportion girls	50.1%	51.2%	46.9%
European ancestry, proportion	60.9%	63.7%	55.1%
Prenatal stress score^a^, mean (SD), range	0.50 (0.41), 0–2.55	0.57 (0.46), 0–2.44	2.03 (1.40), 0–7.00
Postnatal stress score^a^, mean (SD)	0.73 (0.47), 0–3.30	0.74 (0.45), 0–2.53	1.13 (1.69), 0–20.00
Subcortical brain volumes, mean (SD), mm^3^			
Accumbens	1364.40 (188.42)	1356.90 (191.69)	1177.22 (182.09)
Amygdala	3565.00 (382.75)	3542.00 (385.36)	3168.85 (406.89)
Caudate	8187.00 (952.00)	8116.00 (1014.96)	8152.82 (1007.34)
Hippocampus	8074.00 (716.50)	8058.00 (735.01)	8123.39 (788.78)
Pallidum	3925.00 (407.22)	3914.00 (408.27)	3533.88 (378.91)
Putamen	10,794.00 (1107.30)	10,769.00 (1175.36)	11,715.81 (1254.20)
Thalamus	14,944.00 (1338.46)	14,844.00 (1296.98)	14,937.13 (1355.08)

*Note*: ELS scores are computed differently across Generation R and ABCD cohorts so cannot be compared directly. Abbreviation: ABCD, adolescent brain cognitive development.

a Values are non-standardised.

**Table 2:** Significant genetic, environmental, and gene-environmental interaction effects on child subcortical brain volume

Model and outcome	Marker	Lead SNP	Annotated gene	A1/A2	A1 frequency	Effect	s.e.	*p*
Gmodel								
Accumbens	4:65571003	rs7700011	Intergenic	T/C	0.34	0.1515	0.0276	3.83e^−8^
Accumbens	4:65577112	rs7700011	Intergenic	T/C	0.34	0.1504	0.0275	4.53e^−8^
Accumbens	4:65570147	rs7700011	Intergenic	A/G	0.34	0.1504	0.0276	4.89e^−8^
Emodel-prenatal effects								
Intracranial volume	NA					−0.0771	0.0200	1.12e^−3^
Caudate	NA					−0.0576	0.0202	4.36e^−3^
Emodel-postnatal effects								
Intracranial volume	NA					−0.0982	0.0185	1.19e^−7^
GxEmodel-prenatal effects							
Caudate	4:119103684	rs139505895	*PRSS12;NDST3*	A/G	0.02	0.4456	0.0812	4.02e^−8^
GxEmodel-postnatal effects							
Accumbens	6:33321666	rs3130008	*CUTA;SYNGAP1;TABP*	A/G	0.56	−0.1417	0.0253	2.06e^−8^
Accumbens	11:91535952	rs2397823	Intergenic	T/C	0.22	−0.1794	0.0321	2.28e–^8^

*Note*: Analyses pertaining to sub-cortical brain volumes were adjusted for intracranial volume. **Please note that none of the GWAS and GWEIS analyses survived more stringent correction for number of genome-wide analyses performed in each analysis set (see** Figure S1). Abbreviations: A1 frequency, average effect allele frequency; A1, effect allele; A2, other allele; SNP, single nucleotide polymorphism.

**Table 3:** Association between polygenic score of genetic (left), gene-by-prenatal stress interaction (middle) and gene-by-postnatal stress (right) for all subcortical volumes in the independent ABCD study

*p*-value thresholds	G		GxE prenatal^a^		GxE postnatal^b^	
	*β* (95% CI)	*p*	*β* (95% CI)	*p*	*β* (95% CI)	*p*
Accumbens^c^	0.04 (0.02; 0.05)	4.78e^−5^*	−0.06 (–0.13; 0.01)	0.083	−0.02 (–0.05; 0.01)	0.219
Amygdala^c^	0.03 (0.02; 0.04)	3.11e^−5^*	−0.05 (–0.08; –0.02)	6.89e^−3^	−0.04 (–0.06; –0.01)	5.49e^−3^
Caudate^c^	0.05 (0.03; 0.06)	2.15e^−9^*	0.01 (–0.02; 0.05)	0.500	0.00 (–0.02; 0.03)	0.897
Hippocampus^c^	0.03 (0.01; 0.04)	1.28e^−4^*	−0.01 (–0.06; 0.03)	0.477	−0.02 (–0.07; 0.03)	0.356
Pallidum^c^	0.03 (0.02; 0.05)	6.03e^−5^*	0.07 (0.02; 0.13)	0.012	0.07 (–0.01; 0.14)	0.078
Putamen^c^	0.05 (0.03; 0.06)	1.87e^−5^*	0.05 (–0.02; 0.13)	0.188	0.00 (–0.03; 0.03)	0.975
Thalamus^c^	0.02 (0.01; 0.04)	9.56e^−5^*	−0.06 (–0.09; –0.02)	3.71e^−3^	−0.04 (–0.11; 0.03)	0.259
Intracranial volume	0.03 (0.02; 0.05)	6.37e^−5^*	−0.07 (–0.14; 0.01)	0.074	−0.03 (–0.07; 0.01)	0.127

Note: Analyses are adjusted for sex, age, four principal components of genetic ancestry, scanner type, site, PGS_genotype_ of the corresponding brain volume. * denotes significant after Bonferroni-correction for multiple testing for all 24 tests (i.e, *p* < 0.05/24 = 2.08e^−3^). Abbreviations: ABCD, adolescent brain cognitive development; ELS, early life stress.

a Analyses were additionally adjusted for prenatal ELS.

b Analyses were additionally adjusted for postnatal ELS.

c Analyses were additionally adjusted for intracranial volume.

## Data Availability

The data that support the findings of this study are available on request from the corresponding author. The data are not publicly available due to privacy or ethical restrictions.

## Key points

- Although it is well-established that both genetics and the environment influence brain development, these are typically examined separately.
- In this first genome-wide gene-environment interaction study of brain structure in children, three genetic loci were identified that in interaction with early life stress (ELS) were suggestively associated with subcortical brain volumes. Genes mapped to these loci play a role in brain plasticity and have been implicated in neuro-developmental conditions such as autism spectrum disorders (ASD) and schizophrenia.
- This study lends novel insights into gene-environment interplay on the developing brain. Future child developmental research should prioritise gene-environment interaction research to further explore neuro-developmental pathways of risk from early life onwards.
