# Supplementary material for: Mapping gene by early life stress interactions on child subcortical brain structures: A genome‐wide prospective study
Source: JCPP Adv. 2022 Nov 16;2(4):e12113. doi: 10.1002/jcv2.12113 (PMC7614163; doi:10.1002/jcv2.12113)
Supplement: Supplementary file 1 — Supporting Information S1 [file JCV2-2-e12113-s001.docx]

**Supporting Information**

**Mapping gene by early life stress interactions on child subcortical brain structures: A genome-wide prospective study**

Koen Bolhuis*, Rosa Mulder*, C. Louk de Mol, Serena Defina, Varun Warrier, Tonya White, Henning Tiemeier, Ryan Muetzel, Charlotte Cecil. (* shared first)

**Methods**

**Generation R Study**

*Genotyping*

Genotyping of the Generation R GENR3 sample was performed with the Illumina HumanHap 610 or 660 quad chips, depending on the collection time. A full description of GENR3 processing can be found elsewhere (Medina-Gomez et al., 2015). In short, the genetic data were imputed to the 1000 genomes reference panel (phase 3 version 5). MACH software was used for phasing, Minimac3 software for imputation. Genotyping of the Generation GENR4 sample was performed with the GSA-MD array (version2). The data were imputed to the 1000 genomes reference panel (phase 3 version 5). ShapeIT software was used for phasing, Minimac4 software for imputation. For both GENR3 and GENR4, the genetic data were converted into best-guess genotype calls (uncertainty<0.1). Both samples underwent quality control with PLINK v1.9. Variants with a SNP call rate of <97.5%, with evidence for violation of Hardy-Weinberg equilibrium (*p*<1.00x10^-7^), with a minor allele frequency<1%, or with low imputation quality (R^2^<0.3) were removed. A total of 8914615 autosomal variants for GENR3 and 9350278 autosomal variants for GENR4 (*n*=8579196 in both) were carried forward into further analyses. Samples were excluded in the case of sex mismatches, minimal or excessive heterozygosity (>4 SD of the mean), or a sample call rate of <97.5%. Genetic principal components were obtained using the Hapmap Phase II reference panels (including Northwestern Europeans, Subsaharan West Africans, and Asians) (International HapMap, 2003; International HapMap, 2005). Participants of European ancestry were identified based upon the first four PCs inside the range (<4 SDs of the mean) of the Northwestern European founder population. Similar quality control steps as described above were taken for the European samples of GENR3 and GENR4, resulting in 8018135 and 8315727 autosomal variants, respectively (*n*=7772133 in both).

*Neuroimaging*

Magnetic resonance imaging of the brain was performed with a 3-Tesla GE MR750W magnetic resonance scanner (General Electric, Milwaukee, WI), with an eight-channel head coil (White et al., 2018). Automatic volumetric segmentations of the structural T1-weighted images was performed using the FreeSurfer image analysis software, version 6.0 (Fischl et al., 2004). A full description of the imaging processing and quality assessment has been described previously (Muetzel et al., 2018). In total seven subcortical regions (accumbens, amygdala, caudate, hippocampus, pallidum, putamen, and thalamus) as well as intracranial volume were analysed. Across all subcortical regions, mean levels of left and right hemisphere volumes were computed and corrected for intracranial volume as a covariate, and metrics for subcortical regions and intracranial volume were standardised using Z-score transformation.

*Early life stress (ELS)*

Scores of cumulative exposure to psycho-social risk were calculated to capture a global measure of early life stress (ELS; for a detailed description of the scores’ construction and statistical properties see Defina, 2021, https://github.com/SereDef/cumulative-ELS-score). Two standardised (i.e., with a mean of 0 and a standard deviation of 1) scores were created, corresponding to two developmental periods, i.e. during pregnancy (prenatal) and from birth to age ten years (postnatal). These are composed of five additive risk domains (See Figure below), namely life events (13-15 risks [numbers differ slightly based on whether risk was measured in prenatal of postnatal assessment], e.g., death of parent or pregnancy complications), contextual risk (8-10 risks, e.g., neighbourhood problems, financial difficulties), parental risk (9-11 risks, e.g., parental psychopathology, criminal record), interpersonal risk (11-18 risks, e.g., family conflict, loss of a friend), and direct victimisation (8 risks, only available postnatally, e.g., bullying exposure, childhood maltreatment). The individual risk factors (0 = no risk; 1 = risk) were summed to create each domain, and these were in turn summed and standardised to generate weighted additive prenatal and postnatal ELS scores with a mean of 0 and a standard deviation of 1. Higher scores indicate greater cumulative stress, i.e., multiple stressors occurring in the same individual. The scores have been developed and harmonised across multiple cohorts including Generation R and have previously been found to associate with child outcomes including cognitive function and psychiatric risk (Cecil et al., 2014; Rijlaarsdam et al., 2016; Clayborne et al., 2021; Schuurmans et al., 2022).


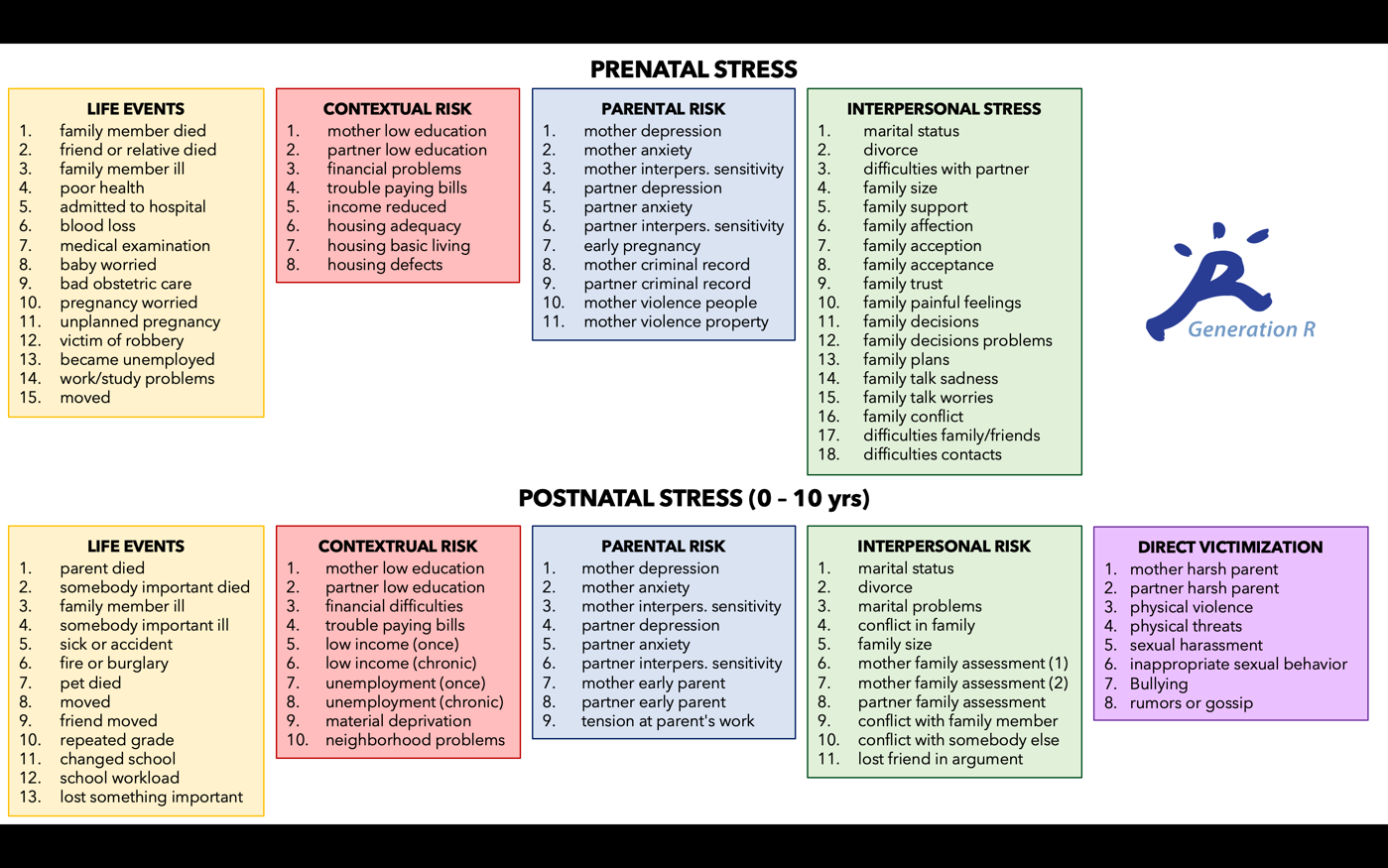


**Score overview**. Overview of all items included in the ELS scores for the Generation R Study. Pre- and postnatal items were assigned to a domain, indicated by the coloured boxes: Life events in yellow, contextual risk in red, parental risk in blue, interpersonal risk in green and direct victimisation in purple. Items were summed to create domain scores, and domain scores in turn were summed and standardised to create total prenatal and postnatal ELS scores.

**ABCD Study**

Supporting analyses were conducted in the independent Adolescent Brain Cognitive Development (ABCD) Study (Casey et al., 2018) as a validation approach, a population-based cohort study including over 10,000 American youth aged 9-10 years. Participants were recruited from 21 sites and imaging methods, assessments, and quality control were harmonised across all sites, which is described in more detail elsewhere (Casey et al., 2018; Nielson et al., 2018).

*Genotyping*

Genotyping of the ABCD sample was performed with the Smokescreen™ Genoyping array (Baurley, Edlund, Pardamean, Conti and Bergen, 2016). The data underwent quality control with Plink v1.9 and were imputed to the 1000 genomes reference panel (phase 3 version 5). ShapeIT software was used for phasing, impute2 software for imputation (Uban et al., 2018). The data were converted into best-guess genotype calls. Variants with a SNP call rate of <95%, with evidence for violation of Hardy-Weinberg equilibrium (*p*<1.00x10^-04^), with a minor allele frequency <1%, or with low imputation quality (Rsq<0.3) were removed. Samples were excluded in the case of sex mismatches, relatedness (Pihat>0.25), minimal or excessive heterozygosity (>2 SD of the mean), or a sample call rate below 95%.

In order to compute polygenic scores, Bayesian regression was first applied to the Generation R meta-analysed GWAS summary statistics of each brain region in order to compute posterior SNP effect sizes under continuous shrinkage priors, using the PRS-CS package (Ge, Chen, Ni, Feng and Smoller, 2019), with the 1KG European reference population under standard settings. PRSice2 software (Choi and O'Reilly, 2019) without clumping or p-value threshold was then applied to compute PGS for each brain region in the ABCD Study. This software takes all genetic variants identified by the GWAS (for the G analysis set) and GWEIS (for the GxE analysis set) in the discovery sample, and calculates a score by weighing the mean number of risk alleles by the SNP effect size. PGSs were standardised to a mean of 0 and a standard deviation of 1 to facilitate interpretation. Higher scores of the PGS_genotype_ reflect higher genetic propensity to larger brain volume. For example, above average scores of PGS_genotype-accumbens_ reflects a higher genetic propensity for larger accumbens volume. The direction of PGS_GxE_ is more challenging to interpret, but higher PGS_GxE_ most likely reflect genetic influences on larger brain volumes in the presence of ELS and/or smaller brain volumes in the absence of ELS (as shown by scatter plots in Figure S3). The PGSs were calculated separately for each subcortical volume outcome as well as separately for GWAS, GWEIS-prenatal stress, and GWEIS-postnatal stress results, resulting in 24 PGSs.

The PGSs were calculated separately for each subcortical volume outcome as well as separately for GWAS, GWEIS-prenatal stress, and GWEIS-postnatal stress results, resulting in three sets of PGS for eight brain outcomes, i.e., 24 PGSs in total. Pearson correlations between all PGS_genotype_, PGS_GxE-prenatal_, PGS_GxE-postnatal_ were calculated in the ABCD Study. For example, correlations were calculated between PGS_genotype-accumbens_ and PGS_GxE-prenatal-accumbens_ and between PGS_genotype-accumbens_ and PGS_genotype-amygdala_ within each cohort separately. Validation analyses proceeded in two steps. First, PGSs were examined in association with their respective brain outcome in the ABCD Study, e.g. the association of PGS_GxE-prenatal_ for the accumbens with accumbens volume. These analyses were adjusted for sex, age, scanner type, site, intracranial volume, and four principal components of genetic ancestry. Analyses using the PGS_GxE_ were additionally adjusted for PGS_genotype_ and cumulative pre- or postnatal ELS scores to parse out the *GxEmodel* effects over and above the effects of *Gmodel* and *Emodel*.

Second, if a significant association was found in the previous analysis step, PGS were next examined to test for associations with later internalising or externalising symptoms, e.g., the association of PGSGxE-prenatal-accumbens with internalising problems., e.g., the association of PGS_GxE-prenatal-accumbens_ with internalising problems. Third, if an association emerged of (a) PGS with brain outcome and (b) PGS with behavioural outcome, statistical mediation was performed to assess whether brain volume mediated the association between the PGS and behaviour outcome. Again, multiple-testing correction was applied using the Bonferroni method.

*Neuroimaging*

Magnetic resonance imaging of the brain was performed using three 3 Tesla scanner platforms: Siemens Prisma, General Electric 750 and Phillips across 21 imaging sites, described in more detail elsewhere (Casey et al., 2018; Nielson et al., 2018). Automatic volumetric segmentations of the same structural T1-weighted images of the subcortical and intracranial volumes took place using FreeSurfer, available for *N*=10,751 participants. As in the Generation R sample, analyses involving subcortical volumes were covaried for intracranial volume.

*Early life stress (ELS)*

Prenatal ELS exposures were assessed with the caregiver-reported ABCD *Developmental History Questionnaire* when the child was on average 9 years old, in line with previous work (Roffman et al., 2021). The following exposures were extracted for each individual and coded as present or absent: unplanned pregnancy; maternal use of alcohol, tobacco, marijuana, cocaine or opioids before pregnancy; maternal use of alcohol, tobacco, marijuana, cocaine or opioids after pregnancy was recognised; Caesarean section; pregnancy complications (e.g. preeclampsia, gestational diabetes); birth complications (e.g., blue at birth, required blood transfusion); and early birth. These exposures were summed to generate a cumulative score of prenatal ELS. In line with a recent study on adverse childhood experiences using ABCD Study data (Karcher, Niendam and Barch, 2020), postnatal ELS exposures were assessed as a sum of parent-rated traumatic experiences of the child from the Kiddie-Structured Assessment for Affective Disorders and Schizophrenia (K-SADS) for DSM-5, a parent-rated question from the K-SADs about whether the child was bullied at school or in the neighbourhood, and seven parent-rated questions of financial adversity from a demographic questionnaire (e.g., “Were evicted from your home for not paying the rent or mortgage?”). Therefore, this definition of postnatal ELS included a number of traumatic life experiences and chronic financial stress. Both ELS scores were scaled to a mean of 0 and a standard deviation of 1.

*Child mental health problems*

Internalising and externalising problems of the child were assessed using the well-validated mother-reported Child Behavior Checklist (CBCL;(Achenbach and Rescorla, 2001)), which was available for *N*=11,063 participants.

**References**

Achenbach, T.A. & Rescorla, L.A. (2001). Manual for the ASEBA School-Age Forms & Profiles. *Burlington, VT: University of Vermont, Research Center for Children, Youth, & Families.*

Baurley, J.W., Edlund, C.K., Pardamean, C.I., Conti, D.V. & Bergen, A.W. (2016). Smokescreen: a targeted genotyping array for addiction research. *Bmc Genomics,* 17.

Casey, B.J., Cannonier, T., Conley, M.I., Cohen, A.O., Barch, D.M., Heitzeg, M.M., [...], Watts, R., Polimeni, J.R., Kuperman, J.M., Fair, D.A., Dale, A.M. & Workgrp, A.I.A. (2018). The Adolescent Brain Cognitive Development (ABCD) study: Imaging acquisition across 21 sites. *Developmental Cognitive Neuroscience,* 32**,** 43-54.

Cecil, C.a.M., Lysenko, L.J., Jaffee, S.R., Pingault, J.B., Smith, R.G., Relton, C.L., Woodward, G., Mcardle, W., Mill, J. & Barker, E.D. (2014). Environmental risk, Oxytocin Receptor Gene (OXTR) methylation and youth callous-unemotional traits: a 13-year longitudinal study. *Molecular Psychiatry,* 19**,** 1071-1077.

Choi, S.W. & O'reilly, P.F. (2019). PRSice-2: Polygenic Risk Score software for biobank-scale data. *Gigascience,* 8.

Clayborne, Z.M., Nilsen, W., Torvik, F.A., Gustavson, K., Bekkhus, M., Gilman, S.E., Khandaker, G.M., Fell, D.B. & Colman, I. (2021). Prenatal maternal stress, child internalizing and externalizing symptoms, and the moderating role of parenting: findings from the Norwegian mother, father, and child cohort study. *Psychological Medicine*.

Fischl, B., Van Der Kouwe, A., Destrieux, C., Halgren, E., Segonne, F., Salat, D.H., Busa, E., Seidman, L.J., Goldstein, J., Kennedy, D., Caviness, V., Makris, N., Rosen, B. & Dale, A.M. (2004). Automatically parcellating the human cerebral cortex. *Cerebral Cortex,* 14**,** 11-22.

Ge, T., Chen, C.Y., Ni, Y., Feng, Y.A. & Smoller, J.W. (2019). Polygenic prediction via Bayesian regression and continuous shrinkage priors. *Nature Communications,* 10**,** 1776.

International Hapmap (2003). The International HapMap Project. *Nature,* 426**,** 789-796.

International Hapmap (2005). A haplotype map of the human genome. *Nature,* 437**,** 1299-1320.

Karcher, N.R., Niendam, T.A. & Barch, D.M. (2020). Adverse childhood experiences and psychotic-like experiences are associated above and beyond shared correlates: Findings from the adolescent brain cognitive development study. *Schizophrenia Research,* 222**,** 235-242.

Medina-Gomez, C., Felix, J.F., Estrada, K., Peters, M.J., Herrera, L., Kruithof, C.J., Duijts, L., Hofman, A., Van Duijn, C.M., Uitterlinden, A.G., Jaddoe, V.W.V. & Rivadeneira, F. (2015). Challenges in conducting genome-wide association studies in highly admixed multi-ethnic populations: the Generation R Study. *European Journal of Epidemiology,* 30**,** 317-330.

Muetzel, R.L., Blanken, L.M.E., Van Der Ende, J., El Marroun, H., Shaw, P., Sudre, G., Van Der Lugt, A., Jaddoe, V.W.V., Verhulst, F.C., Tiemeier, H. & White, T. (2018). Tracking Brain Development and Dimensional Psychiatric Symptoms in Children: A Longitudinal Population-Based Neuroimaging Study. *American Journal of Psychiatry,* 175**,** 54-62.

Nielson, D.M., Pereira, F., Zheng, C.Y., Migineishvili, N., Lee, J.A., Thomas, A.G. & Bandettini, P.A. (2018). Detecting and harmonizing scanner differences in the ABCD study - annual release 1.0. *bioRxiv preprint*.

Rijlaarsdam, J., Pappa, I., Walton, E., Bakermans-Kranenburg, M.J., Mileva-Seitz, V.R., Rippe, R.C., Roza, S.J., Jaddoe, V.W., Verhulst, F.C., Felix, J.F., Cecil, C.A., Relton, C.L., Gaunt, T.R., Mcardle, W., Mill, J., Barker, E.D., Tiemeier, H. & Van, I.M.H. (2016). An epigenome-wide association meta-analysis of prenatal maternal stress in neonates: A model approach for replication. *Epigenetics,* 11**,** 140-149.

Roffman, J.L., Sipahi, E.D., Dowling, K.F., Hughes, D.E., Hopkinson, C.E., Lee, H., Eryilmaz, H., Cohen, L.S., Gilman, J., Doyle, A.E. & Dunn, E.C. (2021). Association of adverse prenatal exposure burden with child psychopathology in the Adolescent Brain Cognitive Development (ABCD) Study. *Plos One,* 16.

Schuurmans, I.K., Luik, A.I., De Maat, D.A., Hillegers, M.H.J., Ikram, M.A. & Cecil, C.A. (2022). The association of early life stress with IQ-achievement discrepancy in children: A population-based study. *Child Development,* in press.

Uban, K.A., Horton, M.K., Jacobus, J., Heyser, C., Thompson, W.K., Tapert, S.F., Madden, P.a.F., Sowell, E.R. & Stu, A.B.C.D. (2018). Biospecimens and the ABCD study: Rationale, methods of collection, measurement and early data. *Developmental Cognitive Neuroscience,* 32**,** 97-106.

White, T., Muetzel, R.L., El Marroun, H., Blanken, L.M.E., Jansen, P., Bolhuis, K., Kocevska, D., Mous, S.E., Mulder, R., Jaddoe, V.W.V., Van Der Lugt, A., Verhulst, F.C. & Tiemeier, H. (2018). Paediatric population neuroimaging and the Generation R Study: the second wave. *European Journal of Epidemiology,* 33**,** 99-125.

Table S1: Pearson correlations between study variables in the GENR3 (below the diagonal) and GENR4 (above the diagonal) subsamples.

|  | ELS-prenatal | ELS-postnatal | Accumbens | Amygdala | Hippocampus | Caudate | Pallidum | Putamen | Thalamus | ICV |
| --- | --- | --- | --- | --- | --- | --- | --- | --- | --- | --- |
| ELS-prenatal | - | 0.62* | -0.09* | -0.08 | -0.09* | -0.16* | -0.06 | -0.02 | -0.14* | -0.19* |
| ELS-postnatal | 0.61* | - | -0.11* | -0.05 | -0.06 | -0.11* | 0.00 | -0.01 | -0.10* | -0.14* |
| Accumbens | -0.10* | -0.13* | - | 0.45* | 0.36* | 0.49* | 0.45* | 0.44* | 0.45* | 0.49* |
| Amygdala | -0.10* | -0.09* | 0.45* | - | 0.69* | 0.46* | 0.52* | 0.53* | 0.54* | 0.59* |
| Hippocampus | -0.11* | -0.09* | 0.42* | 0.67* | - | 0.43* | 0.53* | 0.49* | 0.59* | 0.60* |
| Caudate | -0.15* | -0.14* | 0.48* | 0.42* | 0.41* | - | 0.52* | 0.48* | 0.50* | 0.56* |
| Pallidum | -0.07* | -0.07* | 0.50* | 0.49* | 0.50* | 0.50* | - | 0.67* | 0.67* | 0.61* |
| Putamen | -0.04 | -0.05* | 0.48* | 0.46* | 0.43* | 0.48* | 0.66* | - | 0.51* | 0.54* |
| Thalamus | -0.14* | -0.14* | 0.51* | 0.56* | 0.59* | 0.51* | 0.64* | 0.52* | - | 0.77* |
| ICV | -0.17* | -0.15* | 0.58* | 0.62* | 0.63* | 0.57* | 0.56* | 0.52* | 0.76* | - |

Note: * denotes correlations were statistically significant at *P* < 0.05.

Table S2: Pearson correlations between study variables in the ABCD Study (below the diagonal). Partial correlations adjusted for prenatal or postnatal ELS are shown above the diagonal Note: * denotes correlations were statistically significant at *P* < 0.05.

|  | ELS-prenatal | ELS-postnatal | Accumbens | Amygdala | Hippocampus | Caudate | Pallidum | Putamen | Thalamus | ICV | Int. | Ext. |
| --- | --- | --- | --- | --- | --- | --- | --- | --- | --- | --- | --- | --- |
| ELS-prenatal | - |  | -0.04* | -0.01 | -0.03* | -0.03* | -0.04* | -0.02* | -0.04* | -0.01 | 0.07* | 0.11* |
| ELS-postnatal | 0.22* | - | -0.05* | -0.08* | -0.01* | -0.07* | -0.05* | -0.07* | -0.08* | -0.01* | 0.24* | 0.27* |
| Accumbens | -0.05* | -0.07* | - |  |  |  |  |  |  |  |  |  |
| Amygdala | -0.03* | -0.09* | 0.44* | - |  |  |  |  |  |  |  |  |
| Hippocampus | -0.05* | -0.11* | 0.39* | 0.65* | - |  |  |  |  |  |  |  |
| Caudate | -0.05* | -0.08* | 0.46* | 0.38* | 0.39* | - |  |  |  |  |  |  |
| Pallidum | -0.05* | -0.06* | 0.47* | 0.41* | 0.47* | 0.52* | - |  |  |  |  |  |
| Putamen | -0.04* | -0.08* | 0.48* | 0.52* | 0.51* | 0.47* | 0.56* | - |  |  |  |  |
| Thalamus | -0.06* | -0.09* | 0.40* | 0.54* | 0.63* | 0.52* | 0.56* | 0.54* | - |  |  |  |
| ICV | -0.03* | -0.10* | 0.41* | 0.62* | 0.66* | 0.53* | 0.53* | 0.59* | 0.78* | - |  |  |
| Internalising | 0.13* | 0.25* | -0.01 | 0.01 | 0.02 | 0.01 | 0.02 | 0.02 | 0.03* | 0.03* | - |  |
| Externalising | 0.16* | 0.29* | -0.05* | -0.03* | -0.05* | -0.03* | -0.01 | -0.02* | -0.03* | -0.02* | 0.58* | - |

Table S3: Top twenty hits from the genome-wide association analyses in association with subcortical brain volume variation, ordered by brain region. Two significance levels were set at the standard *P* < 5e^-08^ and the more conservative *P* < 6.25e-9.

| Outcome | Marker | A1/A2 | A2 frequency | Effect | s.e. | P |
| --- | --- | --- | --- | --- | --- | --- |
| Accumbens | 4:65571003 | T/C | 0.34 | 0.1515 | 0.0276 | 3.829e-08 |
| Accumbens | 4:65577112 | T/C | 0.34 | 0.1504 | 0.0275 | 4.527e-08 |
| Accumbens | 4:65570147 | A/G | 0.34 | 0.1504 | 0.0276 | 4.894e-08 |
| Accumbens | 4:65575457 | A/G | 0.66 | -0.1489 | 0.0275 | 5.995e-08 |
| Accumbens | 4:65582585 | T/C | 0.67 | -0.1515 | 0.0281 | 6.748e-08 |
| Accumbens | 4:65578365 | T/C | 0.66 | -0.1478 | 0.0275 | 7.57e-08 |
| Accumbens | 4:65571294 | A/C | 0.66 | -0.1460 | 0.0275 | 1.062e-07 |
| Amygdala | 1:246952424 | A/C | 0.25 | 0.1468 | 0.0271 | 6.31e-08 |
| Amygdala | 10:117437992 | A/G | 0.75 | 0.1484 | 0.0278 | 9.698e-08 |
| Caudate | 6:127097775 | T/C | 0.58 | -0.1354 | 0.0253 | 8.738e-08 |
| Hippocampus | 19:1481559 | T/C | 0.32 | 0.1363 | 0.0250 | 5.056e-08 |
| Hippocampus | 3:88919679 | T/G | 0.17 | -0.1701 | 0.0317 | 8.449e-08 |
| Hippocampus | 3:88901377 | T/C | 0.83 | 0.1690 | 0.0318 | 1.055e-07 |
| Hippocampus | 3:88906785 | A/G | 0.17 | -0.1690 | 0.0318 | 1.055e-07 |
| Hippocampus | 3:88907833 | T/C | 0.83 | 0.1690 | 0.0318 | 1.055e-07 |
| Hippocampus | 3:88909883 | C/G | 0.17 | -0.1690 | 0.0318 | 1.055e-07 |
| Hippocampus | 3:88911404 | A/C | 0.83 | 0.1690 | 0.0318 | 1.055e-07 |
| Hippocampus | 3:88920602 | T/C | 0.17 | -0.1685 | 0.0318 | 1.107e-07 |
| Hippocampus | 3:88920984 | A/C | 0.83 | 0.1685 | 0.0318 | 1.107e-07 |
| Intracranial volume | 14:97592670 | A/G | 0.05 | -0.3020 | 0.0569 | 1.082e-07 |

A1: effect allele, A2: other allele, A2 frequency: average effect allele frequency

Table S4: *Emodel* associations of prenatal (upper panel) and postnatal stress with brain structures.

| Model and brain outcome | Effect | s.e. | P |
| --- | --- | --- | --- |
| *Emodel*: Prenatal stress |  |  |  |
| Accumbens | -0.0105 | 0.0215 | 0.627 |
| Amygdala | 0.0089 | 0.0193 | 0.645 |
| Caudate | -0.0576 | 0.0202 | 4.36e-03 |
| Hippocampus | -0.0032 | 0.0193 | 0.870 |
| Pallidum | -0.0154 | 0.0202 | 0.445 |
| Putamen | 0.0357 | 0.0208 | 0.086 |
| Thalamus | 0.0083 | 0.0160 | 0.604 |
| Intracranial volume | -0.0771 | 0.0200 | 1.12e-04* |
| *Emodel*: Postnatal stress |  |  |  |
| Accumbens | -0.0547 | 0.0201 | 6.42e-03 |
| Amygdala | 0.0000 | 0.0180 | 0.9978 |
| Caudate | -0.0467 | 0.0189 | 0.013 |
| Hippocampus | -0.0034 | 0.0180 | 0.848 |
| Pallidum | 0.0014 | 0.0189 | 0.942 |
| Putamen | 0.0183 | 0.0194 | 0.346 |
| Thalamus | -0.0114 | 0.0150 | 0.446 |
| Intracranial volume | -0.0982 | 0.0185 | 1.19e-07* |

Note: Bonferroni-corrected significance level was set at *P* = 0.00625 (16 tests: 0.05 / 16 = 3.13e-3) and significant associations are marked with an asterisk.

Table S5: Top twenty hits from the genome-wide gene-by-prenatal stress interaction analyses in association with subcortical brain volume variation, ordered by brain region. Two significance levels were set at the standard *P* < 5e^-08^ and the more conservative *P* < 6.25e-9.

| Outcome | Marker | A1/A2 | A1 frequency | Effect | s.e. | P |
| --- | --- | --- | --- | --- | --- | --- |
| Accumbens | 21:18140011 | T/C | 0.55 | 0.1295 | 0.0263 | 8.623e-07 |
| Amygdala | 8:114956725 | A/G | 0.97 | 0.3805 | 0.0704 | 6.337e-08 |
| Caudate | 4:119103684 | A/G | 0.02 | 0.4456 | 0.0812 | 4.02e-08 |
| Caudate | 4:119096261 | A/G | 0.03 | 0.3641 | 0.0682 | 9.517e-08 |
| Caudate | 4:119127814 | A/C | 0.97 | -0.3624 | 0.0682 | 1.059e-07 |
| Caudate | 4:119129047 | C/G | 0.03 | 0.3611 | 0.0682 | 1.179e-07 |
| Caudate | 4:119105988 | A/G | 0.03 | -0.3611 | 0.0682 | 1.179e-07 |
| Caudate | 4:119108256 | A/G | 0.97 | 0.3611 | 0.0682 | 1.179e-07 |
| Caudate | 4:119121676 | CG | 0.03 | 0.3611 | 0.0682 | 1.179e-07 |
| Caudate | 8:139670322 | T/C | 0.21 | 0.1399 | 0.0278 | 4.904e-07 |
| Caudate | 6:129197466 | T/C | 0.07 | -0.2278 | 0.0457 | 6.138e-07 |
| Caudate | 12:55321044 | A/G | 0.21 | 0.1523 | 0.0308 | 7.879e-07 |
| Hippocampus | 2:230744160 | C/G | 0.83 | -0.1486 | 0.0298 | 6.333e-07 |
| Pallidum | 12:4392391 | A/C | 0.47 | -0.1223 | 0.0242 | 4.396e-07 |
| Pallidum | 10:35261292 | A/G | 0.68 | -0.1306 | 0.0265 | 8.196e-07 |
| Pallidum | 8:14678546 | A/T | 0.98 | -0.7353 | 0.1493 | 8.412e-07 |
| Putamen | 15:53963447 | A/C | 0.06 | 0.3238 | 0.0645 | 5.223e-07 |
| Thalamus | 2:46191476 | T/C | 0.93 | 0.2045 | 0.0398 | 2.843e-07 |
| Thalamus | 10:18997606 | T/G | 0.82 | 0.1381 | 0.0275 | 5.223e-07 |
| Thalamus | 5:71132351 | T/C | 0.65 | 0.1014 | 0.0206 | 8.408e-07 |

A1: effect allele, A2: other allele, A2 frequency: average effect allele frequency

Table S6: Top twenty hits from the genome-wide gene-by-postnatal stress interaction analyses in association with subcortical brain volume variation, ordered by brain region. Two significance levels were set at the standard *P* < 5e^-08^ and the more conservative *P* < 6.25e-9.

| Outcome | Marker | A1/A2 | A1 frequency | Effect | s.e. | P |
| --- | --- | --- | --- | --- | --- | --- |
| Accumbens | 6:33321666 | A/G | 0.56 | -0.1417 | 0.0253 | 2.056e-08 |
| Accumbens | 11:91535952 | T/C | 0.22 | -0.1794 | 0.0321 | 2.28e-08 |
| Accumbens | 6:33324527 | T/G | 0.57 | -0.1362 | 0.0254 | 8.018e-08 |
| Accumbens | 10:92708697 | C/G | 0.18 | 0.1759 | 0.0345 | 3.496e-07 |
| Caudate | 10:110363455 | C/G | 0.10 | 0.2090 | 0.0411 | 3.618e-07 |
| Caudate | 10:110363547 | T/C | 0.10 | 0.2090 | 0.0411 | 3.618e-07 |
| Caudate | 10:110363291 | A/G | 0.10 | 0.2081 | 0.0411 | 4.089e-07 |
| Caudate | 1:166518497 | A/T | 0.60 | -0.1273 | 0.0252 | 4.186e-07 |
| Pallidum | 6:34514526 | T/C | 0.03 | 0.3313 | 0.0643 | 2.596e-07 |
| Pallidum | 6:33850861 | A/G | 0.05 | 0.2643 | 0.0513 | 2.641e-07 |
| Pallidum | 7:95503994 | C/G | 0.14 | 0.164 | 0.0328 | 4.246e-07 |
| Pallidum | 9:125112434 | A/G | 0.02 | 0.3724 | 0.0730 | 3.337e-07 |
| Pallidum | 12:4766798 | T/C | 0.02 | 0.3848 | 0.0754 | 3.369e-07 |
| Pallidum | 7:95502146 | T/C | 0.15 | 0.1660 | 0.0326 | 3.612e-07 |
| Pallidum | 6:34514929 | T/C | 0.03 | 0.3019 | 0.0594 | 3.76e-07 |
| Thalamus | 3:106137507 | C/G | 0.96 | -0.2380 | 0.0448 | 1.116e-08 |
| Thalamus | 6:112742649 | T/C | 0.02 | 0.3078 | 0.0581 | 1.169e-07 |
| Thalamus | 7:24085854 | A/G | 0.30 | 0.1104 | 0.0215 | 2.819e-07 |
| Thalamus | 7:24086533 | T/G | 0.71 | -0.1107 | 0.0218 | 3.842e-07 |
| Thalamus | 7:24086346 | T/C | 0.71 | -0.1105 | 0.0218 | 3.842e-07 |

A1: effect allele, A2: other allele, A2 frequency: average effect allele frequency

Table S7: Association between polygenic score of genetic effects for all brain volumes with mother-reported internalising (left) and externalising (right) problems in the independent ABCD Study.

|  | Internalising problems | | Externalising problems | |
| --- | --- | --- | --- | --- |
| Polygenic score | β (95% CI) | *P* | β (95% CI) | *P* |
| Accumbens | 0.00 (-0.02;0.02) | 0.737 | -0.01 (-0.03;0.01) | 0.258 |
| Amygdala | 0.00 (-0.02;0.02) | 0.714 | -0.01 (-0.03;0.01) | 0.378 |
| Caudate | 0.00 (-0.02;0.02) | 0.961 | 0.00 (-0.01;0.02) | 0.612 |
| Hippocampus | 0.00 (-0.02;0.02) | 0.674 | 0.00 (-0.02;0.02) | 0.916 |
| Pallidum | 0.00 (-0.02;0.02) | 0.767 | -0.01 (-0.03;0.01) | 0.572 |
| Putamen | 0.00 (-0.02;0.02) | 0.739 | 0.01 (-0.01;0.03) | 0.668 |
| Thalamus | 0.01 (-0.01;0.03) | 0.470 | 0.00 (-0.02;0.02) | 0.953 |
| Intracranial volume | -0.01 (-0.03;0.01) | 0.187 | -0.02 (-0.04;0.00) | 0.042 |

Note: Analyses are adjusted for sex, age, 4 principal components of genetic ancestry, and research site. * denotes significant after Bonferroni-correction for multiple testing for all 16 tests (i.e., *P* = 0.05 / 16 = 3.13e^-3^).

Table S8: Significant genetic, environmental, and gene-environmental interaction effects on child subcortical brain volumes in the Generation R discovery sample, participants of European genetic ancestry only.

| Model and outcome | Marker | Effect | s.e. | P |
| --- | --- | --- | --- | --- |
| *Gmodel* |  |  |  |  |
| Accumbens | 4:65571003 | -0.1477 | 0.0353 | 2.900e-05 |
| Accumbens | 4:65577112 | -0.1477 | 0.0353 | 2.887e-05 |
| Accumbens | 4:65570147 | -0.1457 | 0.0353 | 3.714e-05 |
| *Environmental – prenatal effects* |  |  |  |  |
| Intracranial volume | NA | -0.0829 | 0.0232 | 3.496e-04 |
| Caudate | NA | -0.0410 | 0.0229 | 7.318e-02 |
| *Environmental – postnatal effects* |  |  |  |  |
| Intracranial volume | NA | -0.1103 | 0.0229 | 1.428e-06 |
| *GxEmodel – prenatal effects* |  | Effect | s.e. | P |
| Caudate | 4:119103684 | -0.2580 | 0.1246 | 3.843e-02 |
| *GxEmodel – postnatal effects* |  |  |  |  |
| Accumbens | 6:33321666 | -0.1842 | 0.0352 | 1.677e-07 |
| Accumbens | 11:91535952 | -0.1429 | 0.0388 | 2.286e-04 |

Table S9: Association between polygenic score of genetic (left), gene-by-prenatal stress interaction (middle) and gene-by-postnatal stress (right) for all subcortical volumes in the independent ABCD Study, participants of European descent only (*n* = 5773).

|  | G | | GxE prenatal | | GxE postnatal | |
| --- | --- | --- | --- | --- | --- | --- |
| P-value thresholds | β (95% CI) | *P* | β (95% CI) | *P* | β (95% CI) | *P* |
| Accumbens | 0.05 (0.03;0.07) | 2.87e^-6^ | 0.01 (-0.01;0.03) | 0.256 | -0.01 (-0.03;0.01) | 0.447 |
| Amygdala | 0.03 (0.02;0.05) | 3.74e^-4^ | -0.02 (-0.04;-0.01) | 0.013 | -0.01 (-0.03;0.00) | 0.135 |
| Caudate | 0.02 (0.00;0.05) | 0.030 | 0.01 (-0.01;0.03) | 0.291 | 0.01 (-0.01;0.03) | 0.353 |
| Hippocampus | 0.03 (0.01;0.05) | 0.011 | 0.00 (-0.02;0.02) | 0.870 | 0.00 (-0.02;0.03) | 0.703 |
| Pallidum | 0.01 (-0.01;0.02) | 0.246 | 0.00 (-0.02;0.02) | 0.943 | 0.00 (-0.02;0.02) | 0.814 |
| Putamen | 0.06 (0.04;0.08) | 7.79e^-8^ | 0.00 (-0.02;0.02) | 0.791 | -0.01 (-0.03;0.02) | 0.607 |
| Thalamus | 0.01 (0.00;0.03) | 0.078 | -0.01 (-0.03;0.01) | 0.275 | 0.00 (-0.02;0.02) | 0.878 |
| Intracranial volume | 0.01 (-0.01;0.03) | 0.182 | 0.00 (-0.02;0.02) | 0.826 | -0.02 (-0.04;0.00) | 0.024 |

Note: values are shown for associations at the P-value threshold 0.5. Analyses are adjusted for sex, age, 4 principal components of genetic ancestry, scanner type and scanner site.

Figure S1 Graphical overview of the analysis steps


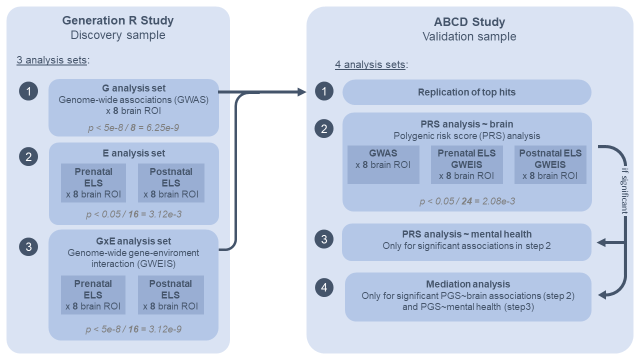


Figure S2: Interaction graph for the GWEIS-significant finding in the independent ABCD Study.


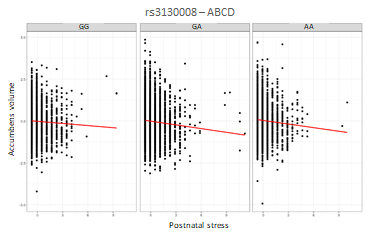


Note: Scatter plot shows differential associations of postnatal stress with accumbens volume for the GG, GA, and AA alleles of the rs3130008 locus (β = 0.0194, se = 0.0108, *P* = 0.0719).

Figure S3: Scatter plots for the association between PGS_GxE_ and subcortical brain volume


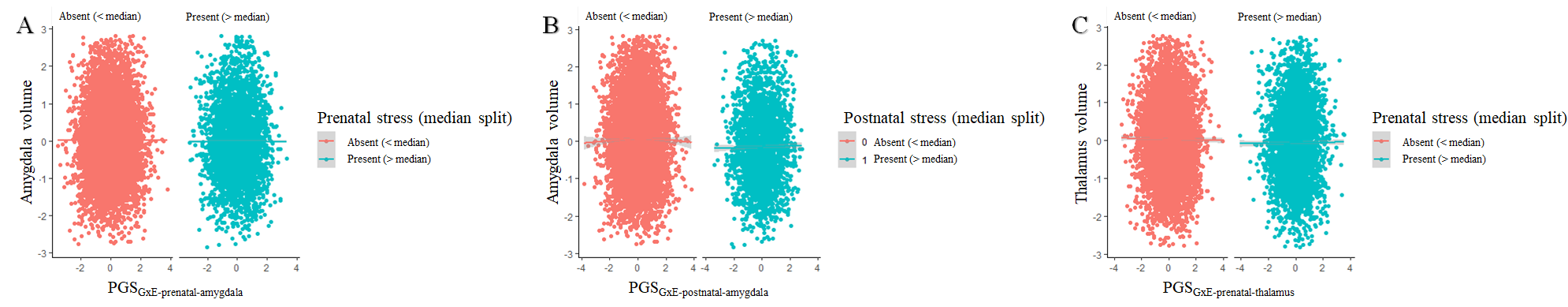


Panel A shows the association between PGS_GxE-prenatal-amygdala_ and amygdala volume, stratified by presence vs. absence of prenatal stress (dichotomised at a median split). Panel B shows the association between PGS_GxE-postnatal-amygdala_ and amygdala volume, stratified by presence vs. absence of postnatal stress (dichotomised at a median split). Panel C shows the association between PGS_GxE-prenatal-thalamus_ and thalamus volume, stratified by presence vs. absence of prenatal stress (dichotomised at a median split).
